# Supplementary material for: Cytomegalovirus protein m154 perturbs the adaptor protein-1 compartment mediating broad-spectrum immune evasion
Source: eLife. 2020 Jan 13;9:e50803. doi: 10.7554/eLife.50803 (PMC6957316; doi:10.7554/eLife.50803)
Supplement: Supplementary file 1. [file elife-50803-supp1.docx]

Supplementary File 1: Key Resources Table

| Reagent type (species) or resource | Designation | Source or reference | Identifiers | Additional information |
| --- | --- | --- | --- | --- |
| antibody | anti-m154 (MCMV) (clone m154.4.113)  mouse monoclonal | (Zarama et al., 2014) | 1mg/ml stock | IF,FC(1:50)  WB (1:200) |
| antibody | anti-m123/IE1 (MCMV) (clone IE1.01)  mouse monoclonal | The in-house antibody production facility of Centre for Proteomics, Rijeka, Croatia | Cat# HR-MCMV-12  1mg/ml stock | IF,FC (1:100)  WB (1:1000) |
| antibody | anti-m20.1 (MCMV) (clone m20.1.02)  mouse monoclonal | (Lenac Rovis et al., 2006) | 1mg/ml stock | IF (1:100) |
| antibody | anti-m06 (MCMV)  (clone croma229)  mouse monoclonal | The in-house antibody production facility of Centre for Proteomics, Rijeka, Croatia | Cat# HR-MCMV-02  1mg/ml stock | FC (1:100) |
| antibody | anti-m04 (MCMV) (clone m04.17)  mouse monoclonal | The in-house antibody production facility of Centre for Proteomics, Rijeka, Croatia | 1mg/ml stock | FC (1:100) |
| antibody | PE anti-pp89 (MCMV)  mouse monoclonal | National Institutes of Health, USA |  | FC(1:50) |
| antibody | anti-mouse CD155 (clone mPVR.01 & .07)  rat monoclonal | This manuscript,  The in-house antibody production facility of Centre for Proteomics, Rijeka, Croatia | 1mg/ml stock | IF,FC (1:100)  WB (1:1000) |
| antibody | anti-mouse CD155 (clone 3F1)  rat monoclonal | Hycult Biotech, Uden, The Netherlands | Cat# HM1106 | IF,FC (1:100)  WB (1:500) |
| antibody | anti-human CD155 (clone hPVR.16)  mouse monoclonal | The in-house antibody production facility of Centre for Proteomics, Rijeka, Croatia | 1mg/ml stock | IF (1:100) |
| antibody | anti-mouse CD229 (Ly9) (clone 7.144.2)  mouse monoclonal | (Sintes et al., 2013) | 1mg/ml stock | IF,FC (1:50) |
| antibody | AF4888 anti-mouse CD48  armenian hamster mono. | Biolegend, San Diego, CA, USA | Cat# 103405 | FC (1:100) |
| antibody | Biotin anti-mouse CD47  mouse monoclonal | Biolegend, San Diego, CA, USA | Cat# 127505 | FC (1:100) |
| antibody | anti-mouse CD18 (clone M18/2)  rat monoclonal | Biolegend, San Diego, CA, USA | Cat# 101402 | FC (1:100) |
| antibody | anti-mouse CD54  mouse monoclonal | Immunotools,  Friesoythe, Germany | Cat# 21279544 | FC (1:100) |
| antibody | anti-mouse CD84 (clone 7)  armenian hamster mono. | Biolegend, San Diego, CA, USA | Cat# 122805 | FC (1:100) |
| antibody | anti-mouse CD162  rat monoclonal | BD Biosciences, San Jose, CA, USA | Cat# 555306 | FC (1:100) |
| antibody | PE anti-mouse CD262 (clone MD5-1)  armenian hamster mono. | eBioscience, San Diego, CA, USA | Cat# 12-5883-82 | FC (1:100) |
| antibody | APC anti-mouse CD270  armenian hamster mono. | Biolegend, San Diego, CA, USA | Cat# 136305 | FC (1:100) |
| antibody | anti-mouse MULT-1 (clone 1D6)  rat monoclonal | The in-house antibody production facility of Centre for Proteomics, Rijeka, Croatia | 1mg/ml stock | FC (1:100) |
| antibody | anti-mouse CD112 (clone 502-57)  rat monoclonal | Hycult Biotech, Uden, The Netherlands | Cat# HM1052 | FC (1:100) |
| antibody | anti-mouse Rae-1ε  rat monoclonal | R&D Systems, Minneapolis, MN, USA | Clone 205001 | FC (1:100) |
| antibody | mouse IgG1 (clone MOPC-21)  mouse monoclonal | BD Pharmingen, San Jose, CA, USA | Cat# 556648 | FC (1:100) |
| antibody | anti-mouse CD80 (clone MEM-233)  mouse monoclonal | Immunotools,  Friesoythe, Germany | Cat# 21270806 | FC (1:100) |
| antibody | APC anti-mouse IFNγ (clone XMG1.2)  rat monoclonal | BD Biosciences, San Jose, CA, USA | Cat# 554413 | FC (1:100) |
| antibody | PE/Cy7 anti-mouse CD8a (clone 53-6.7)  rat monoclonal | eBioscience, San Diego, CA, USA | Cat# 25-0081-82 | FC (1:100) |
| antibody | anti-mouse CD16/CD32 (clone 2.4G2)  rat monoclonal | (Yokoyama and Kim, 2008) |  | FC (1:100) |
| other | AF780 Fixable Viability Dye | eBioscience, San Diego, CA, USA | Cat# 65-0865-14 | FC (1:100) |
| antibody | normal rabbit IgG | Santa Cruz Biotechnology, Dallas, TX, USA | Cat# sc-3888 | IF (1:100) |
| antibody | TRITC anti-rat IgG, F(ab’)2  goat polyclonal | Santa Cruz Biotechnology, Dallas, TX, USA | Cat# sc-3829 | IF (1:200) |
| antibody | FITC anti-rat IgG, F(ab’)2  goat polyclonal | Santa Cruz Biotechnology, Dallas, TX, USA | Cat# sc-3825 | IF,FC (1:200) |
| antibody | FITC anti-mouse Ig  goat polyclonal | BD Biosciences, San Jose, CA, USA | Cat# 554001 | IF,FC (1:100) |
| antibody | APC anti-mouse IgG, F(ab’)2  goat polyclonal | eBioscience, San Diego, CA, USA | Cat# 17-4010-82 | IF (1:200) |
| antibody | PE anti-mouse Ig  goat polyclonal | BD Biosciences, San Jose, CA, USA | Cat# 550589 | FC (1:100) |
| other | PE Streptavidin | Tonbo Biosciences, San Diego, CA, USA | Cat # 54-4317-U500 | FC (1:100) |
| antibody | FITC anti-rabbit IgG, F(ab’)2  goat polyclonal | Santa Cruz Biotechnology, Dallas, TX, USA | Cat# sc-3839 | IF (1:200) |
| antibody | TRITC anti-rabbit IgG, F(ab’)2  goat polyclonal | Santa Cruz Biotechnology, Dallas, TX, USA | Cat# sc-3841 | IF (1:200) |
| antibody | FITC anti-human  donkey polyclonal | Jackson ImmunoResearch, Philadelphia, PA, USA | Cat# 709-095-098 | FC,IF (1:100) |
| antibody | anti-mouse γ1-adaptin  rabbit polyclonal | Santa Cruz Biotechnology, Dallas, TX, USA | Cat# sc-10763 | IF (1:100) |
| antibody | anti-mouse calnexin  rabbit polyclonal | Sigma-Aldrich, St. Louis, MO, USA | Cat# C4731 | IF (1:100) |
| antibody | anti-actin (clone C4)  mouse monoclonal | Merck Millipore, Burlington, MA, USA | Cat# MAB1501 | WB (1:80000) |
| antibody | anti-HA peroxidase (clone 3F10)  goat polyclonal | Roche, Basel, Switzerland | Cat# 12 013 819 001 | WB (1:1000) |
| antibody | anti-asialo GM1  rat polyclonal | Fujifilm Wako Pure Chemical Corporation, Osaka, Japan | Cat# 986-10001 |  |
| antibody | anti-mouse CD4 (YTS 191.1)  rat monoclonal | Laboratory of Stipan Jonjic, Rijeka, Croatia |  |  |
| antibody | anti-mouse CD8 (YTS 169.4)  rat monoclonal | Laboratory of Stipan Jonjic, Rijeka, Croatia |  |  |
| strain, strain background (*Escherichia coli*) | Escherichia coli, strain GS1783 | (Tischer et al., 2010) |  |  |
| strain, strain background-  Mouse cytomegalovirus | wild type (BAC-derived strain pSM3fr) | (Wagner et al., 1999) |  |  |
| strain, strain background-  Mouse cytomegalovirus | BAC-derived strain pSM3fr-GFP | (Mathys et al., 2003) |  |  |
| strain, strain background-  Mouse cytomegalovirus | mutant ∆m144-m158-GFP | (Brune et al., 2006) |  |  |
| strain, strain background-  Mouse cytomegalovirus | mutant ∆m144-m148-GFP | (Brune et al., 2006) |  |  |
| strain, strain background-  Mouse cytomegalovirus | mutant ∆m149-m153-GFP | (Brune et al., 2006) |  |  |
| strain, strain background-  Mouse cytomegalovirus | mutant ∆m153-m158-GFP | (Brune et al., 2006) |  |  |
| strain, strain background-  Mouse cytomegalovirus | mutant Δm154 | (Zarama et al., 2014) |  |  |
| strain, strain background-  Mouse cytomegalovirus | mutant Δm154*Int* | (Zarama et al., 2014) |  |  |
| strain, strain background-  Mouse cytomegalovirus | mutant m154-DDAA | This manuscript, Laboratory of Stipan Jonjic, Rijeka, Croatia |  |  |
| strain, strain background-  Mouse cytomegalovirus | mutant Δm138/fcr-1 | (Crnkovic-Mertens et al., 1998) |  |  |
| strain, strain background-  Human cytomegalovirus | HCMV strain TB40/E | (Sinzger et al., 2008) |  |  |
| other | TIGIT-Fc, ectodomain of mouse TIGIT, human IgG1 Fc fragment | The in-house antibody production facility of Centre for Proteomics, Rijeka, Croatia | 1mg/ml stock | FC (5 ul per test) |
| other | DNAM-1-Fc, ectodomain of mouse DNAM-1, human IgG1 Fc fragment | The in-house antibody production facility of Centre for Proteomics, Rijeka, Croatia | 1mg/ml stock | FC (5 ul per test) |
| other | h-Fc, human IgG1 Fc fragment | The in-house antibody production facility of Centre for Proteomics, Rijeka, Croatia | 1mg/ml stock | FC (5 ul per test) |
| other | mCD155-Fc, ectodomain of mouse CD155, human IgG1 Fc fragment | The in-house antibody production facility of Centre for Proteomics, Rijeka, Croatia | 1mg/ml stock | FC (5 ul per test) |
| other | pp89-derived peptide (IE1) (MCMV) | JPT Peptide Technologies, Berlin, Germany |  |  |
| chemical compound, drug | Brefeldin A | eBioscience, San Diego, CA, USA | Cat# 00-4506-51 | 1 µg/ml |
| chemical compound, drug | Pierce EZ-LINK Sulfo-NHS-SS-biotin labeling kit | Thermo Fischer Scientific, Waltham, MA, USA | Cat# A39258 |  |
| chemical compound, drug | Leupeptin | Sigma-Aldrich, St. Louis, MO, USA | Cat#B7651 | 75ug/ml |
| chemical compound, drug | DAMP | Molecular probes, Eugene, OR, USA | Cat#D-1552 |  |
| cell line (*M. musculus*) | B12 | (Del Val et al., 1991) |  |  |
| cell line (*M. musculus*) | DC2.4 | Merck Millipore, Burlington, MA, USA | Cat# SCC142 |  |
| cell line (*Homo-sapiens*) | HEK 293T cell line | ATCC, Manassas, VA, USA | Cat# CRL-3216 |  |
| strain, strain background  (*M. musculus*) | MAXI mice | (Torti et al., 2011) |  |  |
| strain, strain background  (*M. musculus*) | BALB/c mice | Central Animal Facility, Faculty of Medicine, University of Rijeka and Central Animal Facility, Faculty of Medicine, University of Barcelona, Spain |  |  |
| strain, strain background  (*M. musculus*) | C57BL/6 mice | Central Animal Facility, Faculty of Medicine, University of Rijeka, Croatia |  |  |
| Sequence-based reagent | m154-CTermDelFlag-F: 5'-TCACCGTTGTGATTTTATCTGGGATCGCCGCGGGAGTACTCCTGATCACATACCCATACGATGTTCCAGATTACGCTTGAAGGATGACGACGATAAGTAGGG-3' | Biomers, Ulm, Germany |  |  |
| Sequence-based reagent | m154-CtermDelFlag-R: 5'-AAACACCGCACCAGAGACCAAGTATAAAGCAGTTTTATTGAGCTGATGAGTCAAGCGTAATCTGGAACATCGTATGGGTATGTGATCAGGAGTGTATATCTGGCCCGTACATCGATCT-3' | Biomers, Ulm, Germany |  |  |
| Sequence-based reagent | m154-CTermDelFlag-short-F: 5'-TCACCGTTGTGATTTTATCT-3' | Biomers, Ulm, Germany |  |  |
| Sequence-based reagent | m154-CtermDelFlag-short-R: 5'-AAACACCGCACCAGAGACCA-3' | Biomers, Ulm, Germany |  |  |
| Sequence-based reagent | Pep-Kan-F: 5'-AGGATGACGACGATAAGTAGGG-3' | Biomers, Ulm, Germany |  |  |
| Sequence-based reagent | Pep-Kan-R: 5'-GTATATCTGGCCCGTACATCGATCT-3' | Biomers, Ulm, Germany |  |  |
| Sequence-based reagent | ORF-Kan-F: 5'-ATGAGCCATATTCAACGGGA-3' | Biomers, Ulm, Germany |  |  |
| Sequence-based reagent | ORF-Kan-R: 5'-CTCATCGAGCATCAAATGAAA-3' | Biomers, Ulm, Germany |  |  |
| Sequence-based reagent | m154-AA-Flag-F: 5'-GATCGCCGCGGGAGTACTCCTGATCACACACCGTTGGGAAGCAGCAAAGGGTGGGGAGGTGGCACTCGGGGAAGGTTATGACGAGTCTTATGTGTACCCATACGATGTTCCAGATTACGCTTGAAAGGATGACGACGATAAGTAGGG-3' | Biomers, Ulm, Germany |  |  |
| Sequence-based reagent | m154-AA-Flag-R: 5'- AAACACCGCACCAGAGACCAAGTATAAAGCAGTTTTATTGAGCTGATGAGTCAAGCGTAATCTGGAACATCGTATGGGTACACATAAGACTCGTCATAACCTTCCCCGAGTGCCACCTCCCCACCCTTTGCTGCGTATATCTGGCCCGTACATCGATCT-3' | Biomers, Ulm, Germany |  |  |
| Sequence-based reagent | m154-AA-Flag-F-short: 5'- GATCGCCGCGGGAGTACTCC-3' | Biomers, Ulm, Germany |  |  |
| Sequence-based reagent | m154-AA-Flag-R-short: 5'- AAACACCGCACCAGAGACCAA-3' | Biomers, Ulm, Germany |  |  |
| Sequence-based reagent | m154-HA-R-long: 5’-AAACACCGCACCAGAGACCAAGTATAAAGCAGTTTTATTGAGCTGATGAGTCAAGCGTAATCTGGAACATCGTATGGGTACACATAAGACTCGTATATCTGGCCCGTACATCGATCT-3’ | Biomers, Ulm, Germany |  |  |
| Sequence-based reagent | KanF: 5’-AAGGATGACGACGATAAGTAGGG-3’ | Biomers, Ulm, Germany |  |  |
| Sequence-based reagent | m154-HA-F-long: 5’-ATAAGGGTGGGGAGGTGGCACTCGGGGAAGGTTATGACGAGTCTTATGTGTACCCATACGATGTTCCAGATTACGCTTGAAAGGATGACGACGATAAGTAGGG-3’ | Biomers, Ulm, Germany |  |  |
| Sequence-based reagent | m154-HA-short-R: 5’-AAACACCGCACCAGAGACCAAG-3’ | Biomers, Ulm, Germany |  |  |
| Recombinant DNA reagent | plasmid pIRES Puro | (Glasner et al., 2012) |  |  |
| Recombinant DNA reagent | MCMV BAC pSM3fr | (Wagner et al., 1999) |  |  |
| other | Flow cytometer/cell sorter | BD Biosciences, San Jose, CA, USA | FACSAriaIlu |  |
| other | Flow cytometer | BD Biosciences, San Jose, CA, USA | FACScan |  |
| other | Confocal microscope | Olympus, Tokio, Japan | FV300 |  |
| other | Chemiluminescent imaging system | GE Healthcare, , Chicago, IL, USA | ImageQuant4000 mini |  |
| other | Liquid chromatography system | GE Healthcare, , Chicago, IL, USA | AKTA Purifier |  |
| other | platform for absorption/  luminescence/fluorescence | Berthold, Bad Wildbad, Germany | TriStar |  |
| Software, algorithm | FlowJo_V10 | Tree star, Ashland, OR, USA | RRID:SCR_008520 |  |
| Software, algorithm | GraphPad Prism 8 | GraphPad, San Diego, CA, USA | RRID:SCR_002798 |  |
| Software, algorithm | DiVa v8.0.2. | BD Biosciences, San Jose, CA, USA | RRID:SCR_001456 |  |
| Software, algorithm | FluoView FV300 | Olympus, Tokio, Japan | RRID:SCR_017015 |  |

**References**

Brune W, Wagner M, Messerle M. Manipulating cytomegalovirus genomes by BAC mutagenesis: strategies and applications. In: Reddehase MJ, editor. Cytomegaloviruses Molecular Biology and Immunology. Wymondham: Caister Academic Press; 2006. p. 63-89.

Crnkovic-Mertens I, Messerle M, Milotic I, Szepan U, Kucic N, Krmpotic A, et al. Virus attenuation after deletion of the cytomegalovirus Fc receptor gene is not due to antibody control. Journal of virology. 1998;72(2):1377-82.

Del Val M, Schlicht HJ, Ruppert T, Reddehase MJ, Koszinowski UH. Efficient processing of an antigenic sequence for presentation by MHC class I molecules depends on its neighboring residues in the protein. Cell. 1991;66(6):1145-53.

Glasner A, Zurunic A, Meningher T, Lenac Rovis T, Tsukerman P, Bar-On Y, et al. Elucidating the mechanisms of influenza virus recognition by Ncr1. PloS one. 2012;7(5):e36837.

Lenac Rovis T, Kucan Brlic P, Kaynan N, Juranic Lisnic V, Brizic I, Jordan S, et al. Inflammatory monocytes and NK cells play a crucial role in DNAM-1-dependent control of cytomegalovirus infection. The Journal of experimental medicine. 2016;213(9):1835-50.

Mathys S, Schroeder T, Ellwart J, Koszinowski UH, Messerle M, Just U. Dendritic cells under influence of mouse cytomegalovirus have a physiologic dual role: to initiate and to restrict T cell activation. The Journal of infectious diseases. 2003;187(6):988-99.

Sintes J, Cuenca M, Romero X, Bastos R, Terhorst C, Angulo A, et al. Cutting edge: Ly9 (CD229), a SLAM family receptor, negatively regulates the development of thymic innate memory-like CD8+ T and invariant NKT cells. Journal of immunology. 2013;190(1):21-6.

Sinzger C, Hahn G, Digel M, Katona R, Sampaio KL, Messerle M, et al. Cloning and sequencing of a highly productive, endotheliotropic virus strain derived from human cytomegalovirus TB40/E. The Journal of general virology. 2008;89(Pt 2):359-68.

Tischer BK, Smith GA, Osterrieder N. En passant mutagenesis: a two step markerless red recombination system. Methods in molecular biology. 2010;634:421-30.

Torti N, Walton SM, Brocker T, Rulicke T, Oxenius A. Non-hematopoietic cells in lymph nodes drive memory CD8 T cell inflation during murine cytomegalovirus infection. PLoS pathogens. 2011;7(10):e1002313.

Wagner M, Jonjic S, Koszinowski UH, Messerle M. Systematic excision of vector sequences from the BAC-cloned herpesvirus genome during virus reconstitution. Journal of virology. 1999;73(8):7056-60.

Yokoyama WM, Kim S. Analysis of individual natural killer cell responses. Methods in molecular biology. 2008;415:179-96.

Zarama A, Perez-Carmona N, Farre D, Tomic A, Borst EM, Messerle M, et al. Cytomegalovirus m154 hinders CD48 cell-surface expression and promotes viral escape from host natural killer cell control. PLoS pathogens. 2014;10(3):e1004000.
